# Addressing Gaps in Small-Scale Fisheries: A Low-Cost Tracking System †

**DOI:** 10.3390/s22030839

**Published:** 2022-01-22

**Authors:** Anna Nora Tassetti, Alessandro Galdelli, Jacopo Pulcinella, Adriano Mancini, Luca Bolognini

**Affiliations:** 1 Institute for Marine Biological Resources and Biotechnology (CNR-IRBIM), National Research Council, 60125 Ancona, Italy; annanora.tassetti@cnr.it (A.N.T.); luca.bolognini@cnr.it (L.B.); 2 VRAI Lab, Dipartimento di Ingegneria dell’Informazione, Università Politecnica delle Marche, 60131 Ancona, Italy; a.galdelli@univpm.it (A.G.); a.mancini@univpm.it (A.M.)

**Keywords:** small-scale fisheries, vessel positional data, cloud computing, fishery management, maritime communications

## Abstract

During the last decade vessel-position-recording devices, such as the Vessel Monitoring System and the Automatic Identification System, have increasingly given accurate spatial and quantitative information of industrial fisheries. On the other hand, small-scale fisheries (vessels below 12 m) remain untracked and largely unregulated even though they play an important socio-economic and cultural role in European waters and coastal communities and account for most of the total EU fishing fleet. The typically low-technological capacity of these small-scale fishing boats—for which space and power onboard are often limited—as well their reduced operative range encourage the development of efficient, low-cost, and low-burden tracking solutions. In this context, we designed a cost-effective and scalable prototypic architecture to gather and process positional data from small-scale vessels, making use of a LoRaWAN/cellular network. Data collected by our first installation are presented, as well as its preliminary processing. The emergence of a such low-cost and open-source technology coupled to artificial intelligence could open new opportunities for equipping small-scale vessels, collecting their trajectory data, and estimating their fishing effort (information which has historically not been present). It enables a new monitoring strategy that could effectively include small-scale fleets and support the design of new policies oriented to inform coastal resource and fisheries management.

## 1. Introduction

In large-scale fisheries, the adoption of tracking technologies such as the Vessel Monitoring System (VMS) and the Automated Identification System (AIS) has represented a step toward the application of a more effective ecosystem-based management, contributing to an increase in the information about movements of the fleets [1,2], spatial distribution of fishing grounds, and related fishing pressure [3,4]. Further, vessel positional data and machine-learning analytical algorithms have been used to assess fishing impacts on marine ecosystems [5,6], as well as to support spatial planning and management [7,8] and to monitor compliance to regulations [9,10].

On the other hand, professional small-scale fisheries (SSF), carried out by vessels of an overall length of less than 12 m and not using towed fishing gear [11], are excluded from this scenario even though they may comprise a large part of national fleets and a large number of people employed/dependent on these fisheries [12]. SSF mainly involves the use of traditional passive gears to target multiple resource species [13,14,15] and operates in coastal areas, where many other users considerably compete for space and resources [16] and where sensitive and priority habitats for fisheries are located. The major concern over these fleets is, therefore, their impact on the ecosystem, such as bycatch and discards [17,18,19].

Knowledge of the sector is limited, and information on its fishing effort is sketchy both at national and European Union (EU) level, as VMS is mandatory for fishing vessels from 12 m in length overall and more and AIS from 15 m. Further, the contribution of SSF to fishing mortality of the exploited stocks is underestimated as only vessels above the length of 10 m are obliged to fill in logbooks. This regulatory situation partly explains the lack of SSF information, even though it remains a barrier to understanding the ecological pressures and impacts of small-scale fisheries and their effective management in many places [20].

Limitations in logbook information for the small-scale fleet, as well as in VMS or AIS broadcasts, results in scant possibilities for securing the compliance of the fisheries [21] and in the insufficient reliability of spatial fishing pressure indicators, which, at best, now rely on local approaches that include interviews [22], participatory mapping [23,24], or modeling using generalized behavior rules such as distance from the shore and fishing depths [25,26].

SSF usually cannot accommodate equipment such as VMS or AIS on board for a lack of space or power or simply because, in some cases, it would be too expensive. This encourages the development of alternative low-cost and low-burden tracking solutions.

Only some national initiatives have been implemented in the EU to obtain fine-scale spatio-temporal data from small-scale fishing. They have used AIS, inshore VMS (iVMS; e.g., Marine Institute [27]), Electronic Monitoring (EM) sensors and/or video-based systems, the BlackBox (e.g., Danish Fisheries Agency, [28]), and GPS trackers (e.g., Department for Environment, Fisheries and Rural Affairs, 2018; Marine Scotland, 2019 [29]). Each initiative has revealed different advantages and challenges [21,30].

Similarly, methods to deal with this new highly temporally resolved data have been proposed [31,32], but they are still not harmonized and standardized.

Several members of the International Council for the Exploration of the Sea (ICES) have recently started to discuss appropriate vessel-tracking systems for the whole inshore fleet as well a common framework for identifying trips/hauls using SSF geo-spatial data [33]. Additionally, at the EU level, current negotiations between the EU Commission, Parliament, and Council are underway for the tracking of small-scale fishing vessels by all Member States [34]. Therefore, it is necessary to produce standardized protocols to securely gather and share data across the inshore fleet, classify events during fishing trips (fishing vs. steaming), and infer measures of effort (e.g., number of pots/traps, length of the net, and gear soaking time).

If the use of high-resolution geo-spatial data becomes more wide-spread and standardized, it could fill the major data gaps that exist for the SSF segment.

In this context, an architecture was drafted in Tassetti et al. [35] to collect real-time positional data sent over LoRaWAN or 2G/3G/4G connections by small-scale vessels. Here, it is enriched and tested, and R scripts are released to resolve their individual fishing trips.

The architecture relies on Traccar [36], while the high-tech and cost-efficient Teltonika FMM640 [37] worldwide tracker is proposed as Fleet Management System (FMS) hardware.

Instead of just enforcing small vessels to use other cheaper (e.g., raspberry-based) AIS systems, the choice of this FMS was due to the need to easily share positions and enable monitoring, purchasing inexpensive but robust, well-documented, accessible, and license-free transmitters. This is in line with other ongoing projects [29], which chose similar FMSs.

Moreover, it is worth noting that the use of HTTPS and LoRaWAN technology allows to implement an encrypted communication channel thanks to the TLS/SSL and LoRaWAN protocols, respectively. Both protocols, unlike AIS, enable authentication and encryption of sent data.

A first compact prototype was installed on a small fishing vessel. It records its accurate location, speed, and other features; and sends this data to a dedicated back-end. A sensor attached to the hauler is used in tandem to record when and where it is activated, indicating that the retrieving of the gear is occurring. This information can be assessed in near real time using a secure-access web platform and is recorded allowing future machine-learning analysis and algorithm development in case of vessels that could not be equipped with the hauler sensor.

The coupled use of such low-cost technologies and machine-learning automated analyses opens up the potential for more integrated platforms to inform coastal resource and fisheries management, to support the design and development of new polices, and to understand impacts on the marine ecosystem [3,38].

## 2. Materials and Methods

### 2.1. Monitored Vessel

Currently, the tracking system is installed on an artisanal fishing vessel (10 m in length), operating in the port of Ancona (Italy). The vessel is licensed to use passive gears (gillnets, trammel nets, and traps) and adopts them according to the target species, the market demand, and/or the fishing season. The combination of the above factors inevitably influences the spatio-temporal behavior of the vessel. The use of passive gears includes two distinct operations: the setting and retrieval of the gears, after a certain time period (i.e., soaking time) and based on the above-mentioned factors and sea weather conditions. In addition, specific regulations related to the use of fishing gears are in force, imposing technical/numerical restrictions and regulating the fishing effort exerted at sea [39].

### 2.2. System Architecture

**Figure 1:**
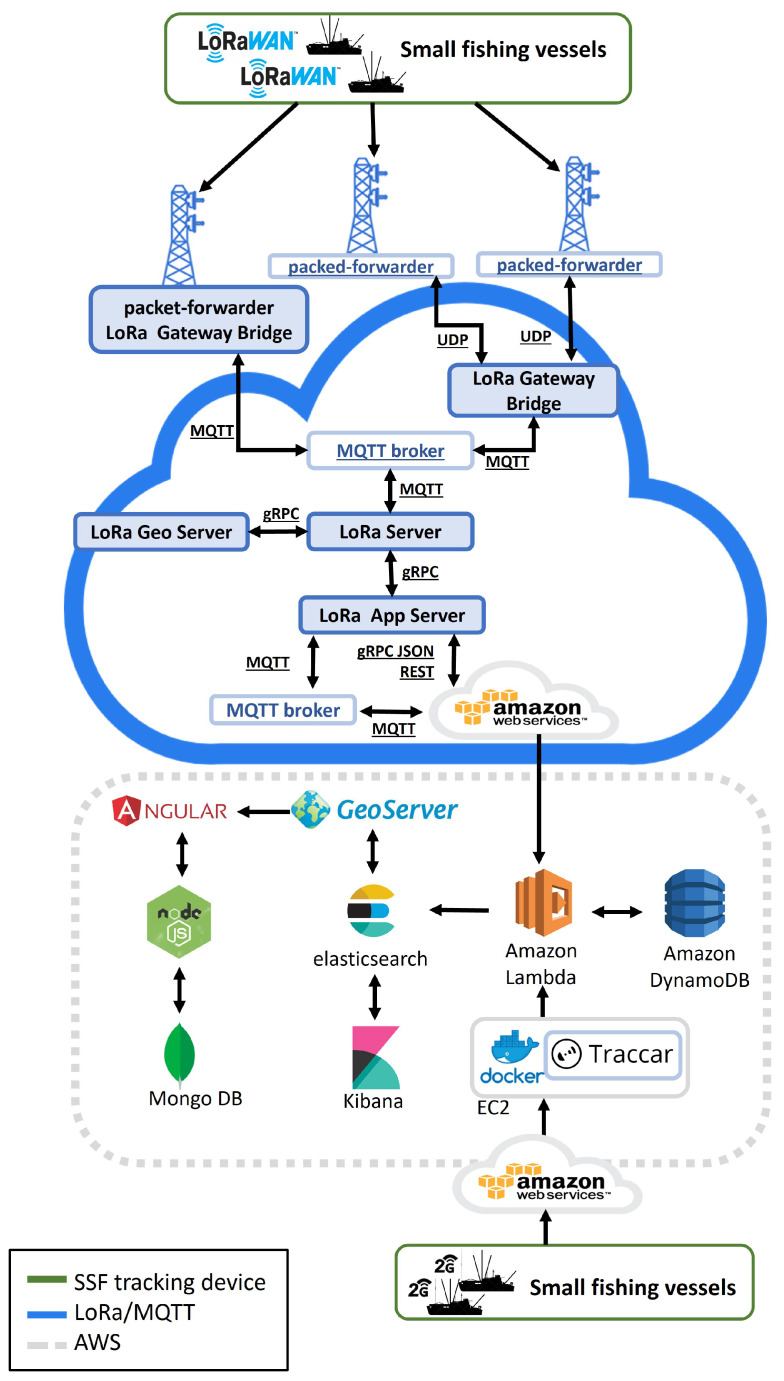
Architecture that manages real-time data sent over LoRaWAN and 2G/3G/4G connections. A GPS tracker collects SSF positional data.

The architecture presented in [40,41] has been adapted to collect and ingest data sent by under-12-meter inshore fishing vessels over two different interfaces. One interface is based on a secure REST API over HTTPS, while another is based on a secure Message Queuing Telemetry Transport Secure (MQTTS) broker (Figure 1). The first interface is dedicated to manage data from 2G/3G/4G devices, while MQTTS is suitable for LoRaWAN broadcasts.

Long Range (LoRa) is an expanded spectrum frequency modulation technique derived from Chirp Spread Spectrum (CSS) technology and is used to implement many Internet of Things (IoT) device networks. The LoRaWAN specification is a Low-Power Wide-Area (LPWA) networking protocol designed to connect IoT devices to the Internet, meeting a number of requirements such as bi-directional communication, end-to-end security, mobility, and localization services. The communication between the LoRa Gateway and the network server is achieved using the MQTT protocol and the MQTT broker as server to collect messages and clients that can read and write to the broker. Different services are used in the LoraWAN infrastructure to manage the requests of several IoT devices. The LoRa Geo Server is used to geolocate LoRaWAN devices, while the Lora App Server provides a web interface where clients, applications, and devices can be managed. In addition, the LoRa App Server is responsible for managing join requests and managing/encrypting application payloads and allows integration with external services thanks to gRPC and RESTful API. Messages collected by the LoRa App server are sent to Amazon Web Services, which in turn forwards the requests to the Amazon Lambda service.

The developed architecture also accepts data from asset trackers such as Teltonika devices (e.g., FMM640) that rely on a mobile network. These trackers send data to the Traccar server through TCP or UDP protocols over secure channels (using TLS/SSL). An interface on a Traccar server is also developed to receive data over a dedicated HTTPS REST interface, which extends the methods available to ingest data. The Traccar server is hosted on the Amazon Elastic Compute Cloud (Amazon EC2) and is managed through Docker. The Traccar system includes a web application—based on the Sencha ExtJS framework and OpenLayers—for managing users, devices, and the map view [42,43].

The set of measured variables is augmented with a proximity inductive sensor (i.e., Omron E2B-M12KN08-WP-B1) that is attached to the hauler.

The processing of this signal enables the detection of fishing activity. A dedicated microcontroller filters the signal of the inductive sensor avoiding anomalous behaviors (e.g., start/stop of hauler) and bouncing (input is debounced). The microcontroller drives a hard line that is acquired by the tracker and then ingested through the above-described architecture. When the hauler is active and rotation is detected by the microcontroller, the output signal is kept high for at least 1 min to ensure that the state is properly sent.

Data are stored in the Amazon DynamoDB database. By using a custom lambda function [44] and basing on events from Traccar, sets of consecutive vessel broadcasts are partitioned into individual fishing trips, which could be retrieved through the dedicated REST API. More details on how trips are reconstructed are reported in the following Section 2.3.

We rely, where possible, on serverless services to ensure a proper scaling when the number of monitor vessels will increase.

Elastic Search and Kibana services are used to retrieve fishing trips in an efficient way while also providing a graphical dashboard for the end-users that can have a quick overview of performed operations over space and time.

Finally, a MongoDB NoSQL database with Node.js and Angular provides the web application to show fishing trips.

**Figure 2:**
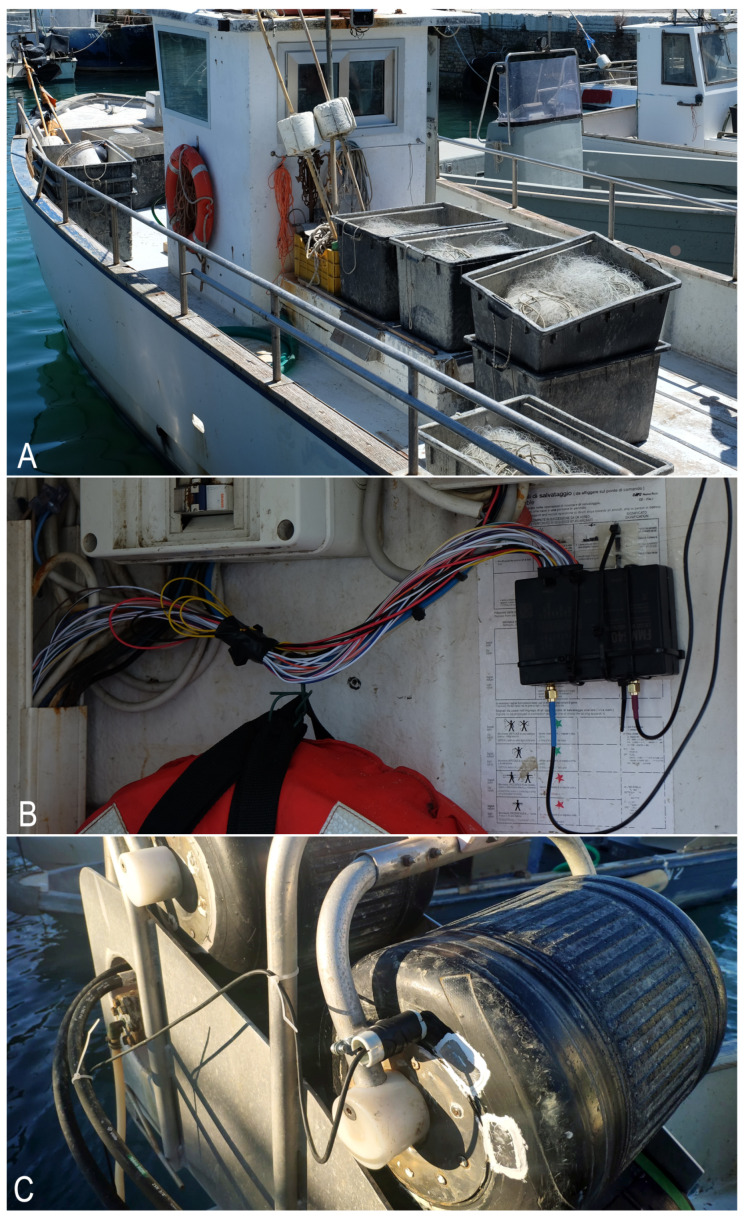
The small-scale fishing vessel (**A**) on board of which the Teltonika FMM640 was installed (**B**) and the proximity inductive sensor attached to the hauler (**C**).

Figure 2 shows the small-scale passive-gear vessel equipped with our first prototype and the speed sensor, which was attached to the hauler and is based on the 4G tracker. In this first installation, the LoraWAN-based solution is indeed enabled but still not implemented.

**Figure 3:**
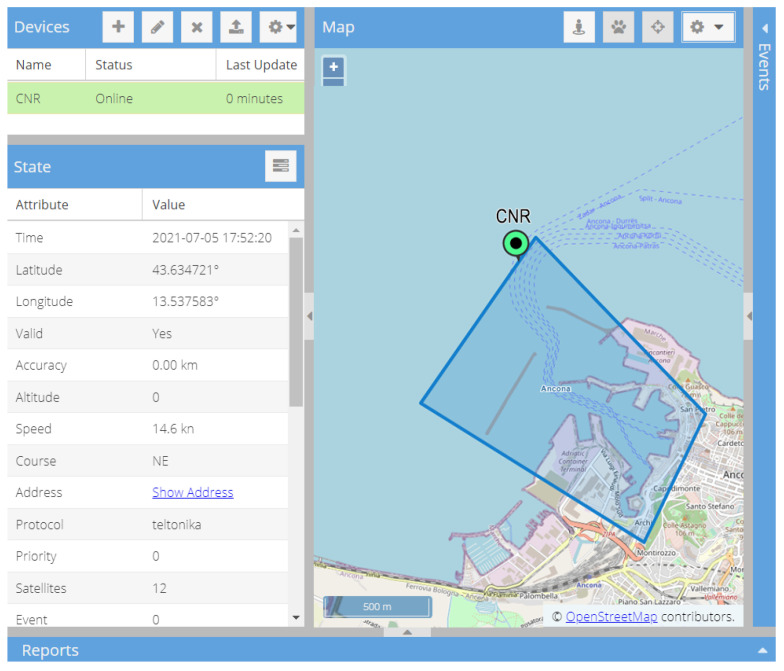
The Web interface of the Sencha ExtJS framework, the equipped small vessel (green point), and the geofence of its homeport (Ancona, Italy). The geofence was defined in Traccar and used in post processing to validate the estimated trips.

Figure 3 refers to the web interface of the Traccar Demo server—based on the Sencha ExtJS framework—that shows the real-time position of the small fishing vessel and the geofence designed to delimit the port and send triggered alerts in real-time.

Table 1 describes the licensing status of the employed technologies.

### 2.3. Data Structure and Processing

GPS positions are progressively ingested according to the data structure reported in Appendix A (Table A1), where *Id* is the progressive serial number, and *deviceId* plays the same role of the Maritime Mobile Service Identities (MMSI) in uniquely identifying vessels. Other fields store position coordinates, timestamps (*deviceTime*), dynamic information relating to the ship’s *course* and *speed*, and system parameters (*sat*, *power*). Geofencing (*type*) registers in real-time when the vessel leaves the harbor and when it enters, while *sensor* is 0 when the hauler is active (and 1 otherwise).

A custom routine was developed in R [45] to partition sequences of consecutive GPS records into individual trips, which starts when the vessel turns on its power and leaves the port and ends on arrival in port (power off). The routine is applied in post processing and implemented in the Amazon Web Services (AWS) architecture. It takes advantage of the events that register the switching on and off of the device as sequences of few subsequent pings with no satellite connection and no battery power. The rationale is to detect trips’ start and end points, by using the over-mentioned zero *sat* and zero *power*—that occurs during device initialization/booting—pings and setting a minimum interval between a shutdown and the subsequent startup of the system. When the boat is stopped for more then a certain amount of time (here 45 min, but it will vary with the fishery), a new trip would start.

The reliability of the system was evaluated for one month (November 2021) in terms of stability of the ping rate and the possible presence of outliers due to errors in data acquisition and transmission (i.e., speed values over 35 km/h and course values not within the range 0–360${}^{\circ }$).

The geofence (Figure 3) was instead used to validate estimated trips, assuming that two geofence events (one *geofenceExit* and one *geofenceEnter*) could define each single trip.

Fishing behavior was investigated in terms of speed values, timestamps, and hauler operations.

Fishing events when the static gear is being recovered are identified using the signal of sensor attached to the hauler, and excluding steaming (i.e., speed values above the 75th quantile). Related fishing positions are plotted to describe the spatial distribution of fishing activity.

Further, as a preliminary investigation of the spatio-temporal behaviour of the vessel, we explore the speed values during the retrieving and setting activities and the amount of time that is spent in the retrieving of the passive gear (i.e., when the hauler is in operation) with respect to the time of day.

GPS sample data and resource code developed for its processing, as well as all the other scripts needed to reproduce the results presented in this article, were written in R and shared [46].

## 3. Results

### 3.1. Data Quality

About 90.5 h of fishing operations were recorded by the tracker during November 2021, corresponding to a regular time series of 5758 records, corresponding to a total of 29 calendar days.

No unrealistic speed or course values were found, and no other errors in device transmission (e.g., spikes and gaps) required interpolation between pings.

### 3.2. Processing and Analysis

A total of 28 trips were identified in November 2021, starting at the moment when the fishing vessel leaves the port and ending on arrival in port. These are reported in Table 2 along with related statistics.

**Figure 4:**
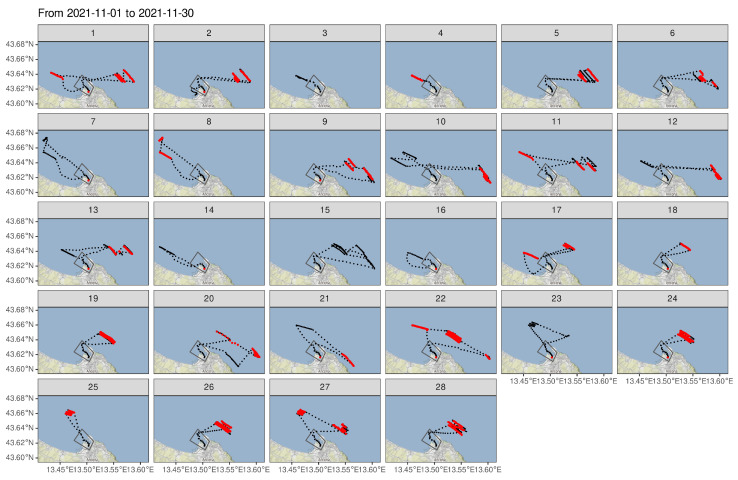
Identified port-to-port trips and sensor activity (red points). The geofence area is represented by the gray solid line.

Maps in Figure 4 highlight the positions where the retrieving of the gear occurs, as revealed by the hauling activity.

The 28 trips were often of few hours (taking a maximum of 4/5 h) and were short hauling operations, characterizing the fishing activity of these vessels with two trips per day. Accordingly, in some trips no hauling activity was detected (Table 2, Figure 4), as the gear was only deployed, and its retrieving was postponed to the following trip. Examples of this include trips 3–4, trips 7–8, and trips 14–19. In other trips due to a mixed behavior of the vessel retrieving, a new setting of the gear can occur in sequence in the same place or in other place, resulting in a more confused spatial pattern of the hauler signals (e.g., trips 10, 11, 12, 20, 21, and 22).

Checking when the vessel leaves and when it enters the geofence, most of the identified trips reported a single enter and exit event. However, in a few trips (e.g., trips 1, 2, 10, 12, and 17), the vessel entered and left the geofence twice to visit fishing grounds set on opposite sides of the designated polygon. It generated false enter/exit events and suggested how the geofence should be reshaped.

**Figure 5:**
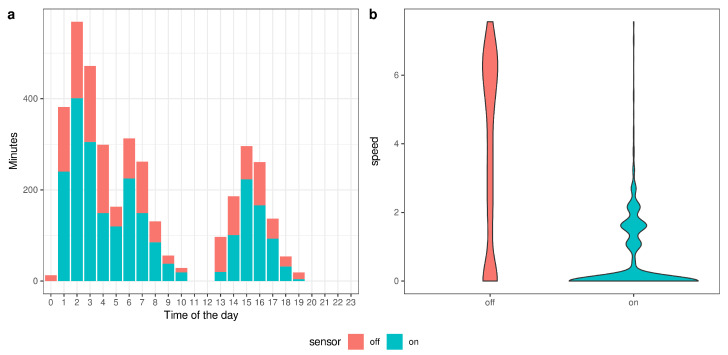
Fishing activity (minutes) over daytime, excluding steaming and in-port reports (**a**), and speed values by hauler activity (**b**).

Hauling activity (i.e., *sensor* on; Figure 5a) indicates that gear was mostly retrieved at night and/or early in the morning (01:00–06:00) and occasionally in the evening.

Speed profiles were strongly affected by hauling activity (Figure 5b), as speed is very low and rarely exceeds 2 km/h while retrieving the gear (i.e., *sensor* on).

## 4. Discussion

Even though small-scale fisheries account for around 75–85 percent of the EU fishing vessels [47,48] and negatively impact marine ecosystems [49], a lack of spatially explicit and quantitative estimates has been a recurrent bottleneck for the application of effective sustainable management [50]. Although some European SSF vessels could meet the costs and technological requirements of AIS or VMS, they do not adopt these technologies as it is not regulated in EU waters. Indeed, in EU fisheries, vessels below 10 m are not required to compile logbooks, and vessels below 12 m are not required to use VMS. Consequently, spatial patterns of small-scale fisheries have been poorly investigated for estimating the pressures and impacts on the ecosystem at high spatial resolution, while coarse spatial indicators of fishing effort have been proposed [51,52]. However, for most SSF vessels, AIS and VMS remain not applicable, due to the low technological capacity and/or implementation costs [53].

The scenario becomes even worse in the Mediterranean and the Black Sea, where SSF makes up the overwhelming majority of the fishing fleet, contributes significantly to food security, and provides valuable employment opportunities, particularly in vulnerable coastal communities [54].

To fill this gap, we deployed a simple tracking equipment on board of a small-scale fishing vessel and coupled this with a serverless and cost-effective architecture to collect data. Currently, hardware costs average nearly EUR 100/150 per unit (and could be reduced by a large volume of purchases), while the service cost is low but related to the number of installed devices (AWS Services Pricing [55]). The actual proposed system works onboard of boats with a storage battery (typically +12 or +24v DC), while the next-generation prototype will be designed for vessels without electrical systems, providing a dedicated battery and solar panel to recharge it. Although new electronic technologies for monitoring small-scale fisheries have been adopted in individual and experimental case studies [21], to our best knowledge, no other similar attempts have been documented in Italy nor in the Mediterranean Sea.

The objective of the proposed system was to minimize costs and license conditions—such those associated with many commercial iVMS and other fleet-tracking systems—and effort by using open-source software and off-the-shelf technologies. This would promote future, large mass adoption, retaining the flexibility to extend system features and improving data collection and analysis. Critical to the choice—and to the future adoption of the system—is also the need to ensure security mechanisms of data transmission and to maintain data confidentiality and encryption. It is indeed important not to underestimate that the majority of fishers understandably prefer to maintain their own data confidentially.

We focused on commercial and relatively inexpensive devices that are widely used in the road-transport sector. They have excellent technical support and firmware already developed to enable the monitoring of vehicle fleets.

The device was fitted directly to the vessel’s ignition system, while a speed sensor was attached to the hauler to signal its activation. For vessels with poor or no power, a solar-powered version of the tracking device will be developed, and the already-proven ability to put the tracker into “hibernation” when not in use will become key to preserving battery power.

It is known that common vessel-tracking technologies such as AIS are not only more expensive and less flexible, but they also may lack coverage [56] and security mechanisms (e.g., terrestrial AIS is an open, non-proprietary, unencrypted, and unprotected radio system that can be picked up by anyone with a receiver). On the other hand, it is worth emphasizing that the proposed solution transmits vessel locations using protocols endowed with security protocols.

Unlike the AIS system, confidentiality is ensured in LoRaWAN and cellular networks through the use of the Public Key Infrastructure (PKI) scheme.

Authenticity and integrity in LoRaWAN are satisfied by design using two session keys encrypted with 128-bit AES. Each packet encrypted with AES-CTR carries a frame counter (to prevent packet replication) and a message integrity code computed with AES-CMAC (to prevent packet tampering) [57,58]. The cellular network, from 3G onwards and using the 3GPP protocol, guarantees data integrity and allows secure authentication thanks to its three main components: user equipment, a mobile management entity, and a home-subscriber server [59]. Instead, AIS provides low levels of authenticity and integrity of transmitted data. While 2G/3G networks suffer from Denial-of-Service (DoS) attacks and an increasing number of subscribers, in 4G communication the availability increases thanks to restrictions on the number of connections into the core network and the new key hierarchy in the Evolved Packet System (EPS) [60].

The availability in LoraWAN and tAIS is lower than in cellular-based solutions (low and medium levels, respectively). Moreover, due to different frequency bands and national regulations, LoRaWAN is not universally compatible [61].

The cost of these technologies is very different (Table 3). LoRaWAN was developed for low-cost IoT solutions of about a few dozen Euros; the cost of cellular technology is in the middle, while both AIS classes cost considerably more.

The experimental results demonstrate that the system guarantees a stable data acquisition ensuring a consistent reconstruction of the fishing patterns of the monitored vessel. In the pre-processing analysis, no errors in the acquisition rate, as well as no transmission gaps or erroneous pings (e.g., no points on land), occurred. Supported by these results, we developed specific routines to define trips and describe related fishing trip behavior.

Different AIS/VMS analyzing methods have been inherited to identify trips using harbor polygons and/or time thresholds [62,63], and they have been promisingly tested with SSF positional data [33]. However, digitizing polygons could become challenging—and related methods of trip reconstruction could fail—particularly when ports have particular shapes (e.g., with approach channels), tracking systems are widely adopted and/or a census of ports does not exist. Moreover, small-scale vessels could leave/return to hot-spots that are not defined as harbors. Already in our case study, defining a wider a priori geofence has made the related alerts useless due to vessel movements near the edge of the polygon.

For such a reason, expecting to equip a large number of vessels operating in different areas, we only relied on system parameters, such as the number of satellites and the power of the battery, to better identify the starting and shutdown of engine ignition and to define trips. This prevents a manual editing/reshaping of harbor polygons and fosters automation of data processing.

The resulting trips allow to draft a preliminary fishing behavior of the monitored vessel that generally performs two trips per day, in line with other studies on small-scale fisheries using passive gear in the study area [15]. Regardless of whether the fisher is using nets or traps, a first trip occurs early in the morning (from 01:00 to 04:00–06:00) and a second trip in the evening (13:00–18:00) depending on the soaking time, which instead is related to the fishing gear in use [15,64].

Further, results highlight how the sensor attached to the hauler is reliable to locate the removal of passive gears, even though an offset error is made between the real rotary motion of the hauler and the transmitted information (due to the timer and the speed at which the token queue fills up). However, improvements in the hauler-acquisition software will be implemented to help in the identification of the gear as well as to guarantee a better estimation of the working time at a high temporal resolution (i.e., seconds).

According to the pattern observed in this work, the vessel retrieved and immediately deployed the gear both in the morning and in the evening, even though there were trips in which the gear was only deployed. However, as shown in Table 2, the timing of this fishing behavior can vary in the short-run (e.g., in a week) due to a number of factors such as the gear in use, the weather conditions, and the season/target species [15]. These should be considered while assessing fishing-effort variables.

Overall, the preliminary results obtained allow to describe fishing patterns in detail but at the same time underlines that there is still a great deal to be done to understand SSF behaviours at sea and to assess related effort. From a fishery-management prospective, collecting SSF data in this way would indeed be particularly effective to support the application of the ecosystem approach to fishery [65,66].

In the framework of the ARGOS Interreg Italy–Croatia Project [67], we expect to monitor the routes of approximately 30 boats, over a period of 3 years, exerting their activity as widely as possible along the Marche Region (Italy). We will properly inform end-users regarding the use of data, and a small financial incentive will be available if a fisher will join our initiative.

The plan is to track different gear types of the fishery (e.g., trammel net, longline, gill net, pots, and traps) and develop machine-learning models to predict when fishing activity occurs when no sensors could be installed to the hauler and estimate measures of fishing effort. To collect ground truths, researchers will make detailed, timed observations of the different vessel activities, including steaming, shooting, and hauling gear, whilst recording GPS tracks. Increasing the number of vessels equipped, we will be able to characterize SSF fishing behaviors in the study area, related fishing grounds, and seasonal exploitation per gear.

Although in recent times monitoring systems based on mobile application represent a tempting and promoted solution [68], especially in those countries where the fishery regulatory system is not more effective [69,70,71], we believe that, where possible, a device directly connected to a vessel engine should allow a more-reliable data acquisition. Indeed, looking toward the application of a legislation that provides for the mandatory monitoring of small-scale fishing vessels, it would be unthinkable to rely on official data collection on personal devices, which can be voluntarily turned off or involuntarily damaged.

## 5. Conclusions

Preliminary results are encouraging as the proposed architecture offers a serverless, cost-effective and low-maintenance solution of data reporting for under-12 metre inshore fishing vessels.

Although processing routines could be improved, also thanks to ongoing monitoring, collected positional data are of fine scale and good quality, as well as the stored individual fishing trips. The small unit seems to be reliable, inexpensive to use (as it has a low-power requirement, operating on 12–24v DC), and easy/quick to fit (as it can be programmed remotely). All this could meet the requirements of government and the fishing industry and could avoid overburdening fishers in terms of reporting.

Moreover, if combined with an appropriate data processing to infer spatially explicit fishing events, such boat tracking could provide sufficient information on SSF fishing activities for sustainable management of the fisheries and the marine ecosystem.

This could enable a new monitoring strategy that effectively includes SSF and supports the design of more efficient policies oriented to inform stakeholders (e.g., fishers themselves, marine planners, and fisheries managers) when making decisions relating to the marine environment and activities [56].

Of course, it is a challenge for fishers and especially for small-scale vessel fishers to adopt and afford new technologies for fisheries monitoring and reporting fishing-activity data. Therefore, in the short future, appropriate incentives and adequate financial compensations will be necessary for integrating these technologies into fisheries-management schemes, and an opportunity could come, for instance, from the European Maritime Fisheries and Aquaculture Fund (EMFAF) [21].

## Figures and Tables

**Table 1:** Licensing/open-access/open-source status of the employed technologies.

Technology	Status
LoRa	Open-access (Lora licensed)
MQTT (Mosquitto)	Open-source (EPL/EDL licensed)
AWS	Open-access (Amazon licensed)
Traccar	Open-source and
	open-access (Traccar licensed)
MongoDB	Open-source (MongoDB license)
NodeJS	Open-source (MIT license)
Angular	Open-source (MIT license)
Kibana,	Open-source (Elastic license 2.0)
elasticsearch	
GeoServer	open-source (Open Source
	Geospatial Foundation license)
Cellular	Mobile Operator licensed
Docker	Open source (Docker licensed)

**Table 2:** Identified trips and related statistics.

Trip	tripStart	tripEnd	Duration (h)	SA * (kmh)	Distance (km)	Hauler (h)	Entry	Exit
1	“2021-11-01 01:40:10”	“2021-11-01 06:50:47”	5.18	4.27	47.27	3.03	2	2
2	“2021-11-03 07:48:34”	“2021-11-03 11:50:37”	4.03	4.28	34.49	1.97	2	1
3	“2021-11-03 15:58:46”	“2021-11-03 16:37:23”	0.64	7.46	9.44	0.00	1	1
4	“2021-11-04 02:46:35”	“2021-11-04 04:02:32”	1.27	3.50	9.59	0.73	1	1
5	“2021-11-04 15:37:07”	“2021-11-04 19:07:55”	3.51	4.44	31.54	1.87	1	1
6	“2021-11-05 02:11:56”	“2021-11-05 05:01:01”	2.82	5.64	31.84	1.32	1	1
7	“2021-11-07 15:07:38”	“2021-11-07 16:34:34”	1.45	8.46	22.51	0.02	1	1
8	“2021-11-08 02:43:32”	“2021-11-08 05:18:12”	2.58	3.96	21.69	1.67	1	1
9	“2021-11-08 06:24:19”	“2021-11-08 10:19:29”	3.92	4.94	39.91	2.13	1	1
10	“2021-11-10 14:18:54”	“2021-11-10 18:32:35”	4.23	6.49	53.53	1.55	2	2
11	“2021-11-11 01:39:33”	“2021-11-11 05:33:34”	3.90	5.12	40.88	2.07	1	1
12	“2021-11-11 15:23:24”	“2021-11-11 18:33:41”	3.17	6.76	40.96	1.15	2	2
13	“2021-11-12 01:43:35”	“2021-11-12 05:39:19”	3.93	4.46	37.66	1.35	1	1
14	“2021-11-12 07:06:50”	“2021-11-12 08:41:23”	1.58	4.87	16.63	0.02	1	1
15	“2021-11-13 01:41:42”	“2021-11-13 06:07:41”	4.43	4.80	45.11	0.00	1	1
16	“2021-11-14 14:59:38”	“2021-11-14 15:56:21”	0.95	6.53	11.92	0.02	1	1
17	“2021-11-15 02:41:28”	“2021-11-15 06:00:27”	3.32	4.00	27.20	1.85	2	2
18	“2021-11-19 06:54:01”	“2021-11-19 08:45:59”	1.87	5.11	18.15	0.62	1	1
19	“2021-11-20 01:58:53”	“2021-11-20 05:37:43”	3.65	3.92	31.12	2.07	1	1
20	“2021-11-20 06:38:39”	“2021-11-20 09:18:39”	2.67	6.46	33.53	1.10	1	1
21	“2021-11-21 14:02:21”	“2021-11-21 17:01:05”	2.98	4.40	26.54	1.35	1	1
22	“2021-11-22 01:45:45”	“2021-11-22 08:09:12”	6.39	3.43	46.42	4.12	1	1
23	“2021-11-24 14:07:27”	“2021-11-24 15:33:19”	1.43	7.86	21.59	0.02	1	1
24	“2021-11-25 01:39:22”	“2021-11-25 06:13:22”	4.57	3.93	38.50	2.48	1	1
25	“2021-11-25 07:28:34”	“2021-11-25 09:13:33”	1.75	5.86	21.09	0.82	1	1
26	“2021-11-25 16:04:59”	“2021-11-25 20:44:41”	4.66	4.37	38.46	2.37	1	1
27	“2021-11-26 14:06:30”	“2021-11-26 18:42:23”	4.60	4.19	38.28	2.42	1	1
28	“2021-11-29 05:13:32”	“2021-11-29 09:47:31”	4.57	3.89	37.49	2.30	1	1

* SA: speed average; Entry/Exit: num. of entry and exit from the geofence.

**Table 3:** Costs of the technological solutions used.

Technology	Cost	
	Hardware	Data Traffic
LoRaWAN	EUR 5–30	-
Cellular	EUR 10–80	EUR cent/KB
AIS	EUR 200–400 (Class B)	-
	EUR 800–5000 (Class A)	-

## Data Availability

Samples of collected data are available at Pulcinella et al. [46], together with the developed R routines. The code is also available at https://github.com/irbimMAPS/ssf (accessed on 14 December 2021).

## Abbreviations

The following abbreviations are used in this manuscript:

AIS	Automated Identification Systems
AWS	Amazon Web Services
EMFAF	European Maritime Fisheries and Aquaculture Fund
EU	European Union
EPS	Evolved Packet System
FMS	Fleet Management System
GPS	Global Positioning System
gRPC	Google Remote Procedure Call
ICES	International Council for the Exploration of the Sea
iVMS	inshore Vessel Monitoring Systems
LoRa	Long Range
LoRaWAN	Long Range Wide Area Network
MQTTS	Message Queuing Telemetry Transport
PKI	Public Key Infrastructure
SSF	Small-scale Fisheries
VMS	Vessel Monitoring Systems
3G/4G	Third/Fourth generation of broadband cellular network

## Appendix A

**Table A1:** Data structure, from the combination of Traccar Server and Teltonika device parameters.

Attribute	Description	Value
id	Ping identification	Number: integer
priority	I/O property type of priority	0–3
sat	Number of satellites	>0
event	System event	0 to 999
sensor	Proximity sensor state	0–1
io22	Current Profile	1 to 5
io71	GNSS status	0—off
		1—no antenna
		2—no fix
		3—got fix
		4—sleep
		5—over current
motion	Motion state	0–1
rssi	Received signal strength indicator	1 to 5
io200	Deep Sleep mode	0 – No Sleep
		1—GPS Sleep
		2—Deep Sleep
		3—Online Sleep
ignition	Ignition state	0–1
battery	Teltonika Battery Voltage	Voltage:mA
io68	Battery Current	Voltage:mA
pdop	Position Dilution of Precision	Number: float
hdop	Horizontal dilution of precision	Number: float
power	Vessel Battery Voltage	Voltage:mA
io24	Speed Over Ground [km/h]	>0
distance	Distance from previous ping	Distance: metres
totalDistance	Odometer	Distance: metres
hours	Hours counter	Hour: ms
deviceId	Device identification	Number: integer
type	Event type	geofenceExit-gefenceEnter
deviceTime	Device time	timestamp
latitude	Latitude	−90${}^{\circ }$ to 90${}^{\circ }$
longitude	Longitude	180${}^{\circ }$ to 180${}^{\circ }$
altitude	Altitude	Altitude: metres
speed	Speed Over Ground [knot]	>0
course	Course Over Ground	−180${}^{\circ }$ to 180${}^{\circ }$
accuracy	Accuracy	Number: integer
